# Supplementary material for: Thinking together: How group argumentation boosts fake news recognition
Source: PLoS One. 2026 May 27;21(5):e0348391. doi: 10.1371/journal.pone.0348391 (PMC13215538; doi:10.1371/journal.pone.0348391)
Supplement: S1 File — Full texts of the four news items and the four order of news presentation and the corresponding types of arguments. (DOCX) [file pone.0348391.s001.docx]

**Supplementary Method**

1. Full texts of the four news items

FORMALDEHYDE*: “Formaldehyde is added to vaccines to kill unwanted bacteria and viruses that could contaminate the vaccine during production. The Center for Disease Control and Prevention ensures that most formaldehyde is removed from the vaccine before it is packaged, but the removal is not complete. Especially in the "younger" vaccines (such as the one developed for COVID-19) and those that have been less tested, the concentration of this aldehyde could be higher. What could the risks be? According to the National Institute of Environmental Health Sciences, formaldehyde is a human carcinogen. There is no evidence to show the existence of a correlation between the amount of formaldehyde present in vaccines and tumor manifestations; however, since it is a question of recent vaccines in the case of COVID-19, the long-term effects are not known”.*

THYROID: *“An American medical program created panic after the statements of the host of the debate, Dr. Mehmet Oz (a surgeon, TV presenter, and American television author of Turkish origin); The doctor, in fact, hypothesized that the increase in cases of thyroid cancer recorded in recent decades is due to the spread of dental radiographs and especially periodic mammography for the early diagnosis of breast cancer. His remarks were picked up by the scientific community in the United States, which unanimously agreed on the requirement to wear a lead collar during radiography to protect the thyroid”.*

NUTELLA: *“Nutella with marijuana exists. It's called Chrontella, and it's produced by a Toronto-based company. It looks like the famous hazelnut cream but has an "extra" ingredient: 300 milligrams of cannabis extract. A jar includes roughly 3 servings and costs a whopping 20 euros. There is no evidence to suggest that it is addictive”.*

MASKS: “*Among the many uncertainties of this pandemic, they all agree on one thing: masks are a very useful weapon to mitigate the spread of the coronavirus. However, their true effectiveness, that is, how much they are able to reduce the amount of viral particles that reach the wearer's respiratory tract, is still unclear. To try to shed some light on the issue today, researchers from the University of Massachusetts Lowell, in collaboration with California Baptist University, showed that surgical masks are an important weapon, but that under certain conditions they can be even more harmful than not wearing them at all. In fact, from their model, it emerged that a mask significantly changes the flow of air around the face: instead of entering the mouth and nose through specific paths, the air enters the mouth and nose through the entire surface of the mask... Therefore, at low percentages of filtering capacity resulting from the repeated use of surgical masks, the number of viral particles that reach the nose and mouth could be even greater than what would happen with the face uncovered. Their study has just been published in the journal Physics of Fluids”*.

1. The four order of news presentation and the corresponding types of arguments

The experimental order matched that of phase 1 and for each group it could be one of the following four:

1) Formaldehyde (individual argumentation) – Nutella (individual argumentation) – Thyroid (group argumentation) – Masks (group argumentation)
2) Nutella (group argumentation) – Formaldehyde (group argumentation) – Masks (individual argumentation) – Thyroid (individual argumentation)

3) Thyroid (individual argumentation) – Masks (individual argumentation) – Formaldehyde (group argumentation) – Nutella (group argumentation)
4) Masks (group argumentation) – Thyroid (group argumentation) – Nutella (individual argumentation) – Formaldehyde (individual argumentation)
