# Supplementary material for: Thinking together: How group argumentation boosts fake news recognition
Source: PLoS One. 2026 May 27;21(5):e0348391. doi: 10.1371/journal.pone.0348391 (PMC13215538; doi:10.1371/journal.pone.0348391)
Supplement: S1 Table — (DOCX) [file pone.0348391.s002.docx]

**S1 Table: pre-test results on news items**

|  | **Correct answers** | **Confidence** | **Clarity of the message** | **interest** |
| --- | --- | --- | --- | --- |
| **News item** | **%** | **Mean (SD)** | **Mean (SD)** | **Mean (SD)** |
| Formaldehyde | 30.4% | 3.87 (1.66) | 5.43 (1.24) | 5.04 (1.94) |
| Thyroid | 65.2% | 3.78 (1.88) | 5.18 (1.30) | 4.73 (1.75) |
| Nutella | 69.6% | 4.48 (1.93) | 5.09 (1.70) | 3.87 (2.18) |
| Masks | 56.5% | 4.87 (1.87) | 5.52 (1.79) | 5.13 (1.79) |
