# Supplementary material for: Thinking together: How group argumentation boosts fake news recognition
Source: PLoS One. 2026 May 27;21(5):e0348391. doi: 10.1371/journal.pone.0348391 (PMC13215538; doi:10.1371/journal.pone.0348391)
Supplement: S2 File — Mixed-effects model for the first fake news item and analysis of the order for real news. (DOCX) [file pone.0348391.s003.docx]

**Supplementary Results**

1. Mixed-effects model for the first fake news item

To examine the robustness of the phase × argumentation type interaction while accounting for the nested structure of the data, we conducted an additional linear mixed-effects analysis. This analysis was restricted to the **first fake news item presented to participants.**

This analytic choice was motivated by our experimental aims. In line with previous work suggesting that insights gained during group discussion may transfer to subsequent reasoning tasks (e.g., Trouche et al., 2014), our study explicitly tested whether discussing a fake news item in a group could influence participants’ later individual evaluations of other fake news items. Because of this design feature, accuracy judgments for the second fake news item could potentially reflect not only the immediate effects of individual versus group argumentation, but also learning or carry-over effects originating from earlier group discussions. Restricting the analysis to the first fake news item therefore allows a more conservative test of the immediate impact of argumentation type, prior to any possible cross-order influence.

We estimated a linear mixed-effects model with accuracy scores as the dependent variable, phase (Phase 1 vs. Phase 2) and type of argumentation (group vs. individual) as fixed effects, and their interaction. Random intercepts were specified for **group (meant as the group to which the participant belonged)**, to account for interdependence among participants within discussion groups. Degrees of freedom were estimated using the Satterthwaite approximation.

The analysis revealed a significant interaction between phase and type of argumentation, F(1, 181) = 4.86, p = .029. Simple effects analyses showed that accuracy significantly increased from Phase 1 to Phase 2 when the fake news item was discussed in groups (estimate = 0.58, SE = 0.21, p = .005), whereas no significant change was observed when the same item was argued individually (estimate = −0.04, SE = 0.20, p = .842). The random intercept for group accounted for a substantial proportion of variance (ICC = .249), indicating meaningful between-group variability.

Overall, this mixed-effects analysis supports the conclusion that the observed improvement in fake news detection following group discussion is robust to group-level interdependence.

1. Analysis of the order for real news

We conducted a repeated measures ANOVA for phase 1 and phase 2 accuracy scores of individually argued real news, with Between Subjects Factor the two different orders for the type of argumentation (*G* VS *I*). In this case, the interaction was not significant (*F*(1, 109) = .038, *p* = .846, η²_p_ = .00).
