# Supplementary material for: Thinking together: How group argumentation boosts fake news recognition
Source: PLoS One. 2026 May 27;21(5):e0348391. doi: 10.1371/journal.pone.0348391 (PMC13215538; doi:10.1371/journal.pone.0348391)
Supplement: S2 Table — (DOCX) [file pone.0348391.s004.docx]

**S2 Table: Linear regression output for emotional/social variables**

|  | | |
| --- | --- | --- |
| **Model** | **R** | **R²** |
| 1 | 0.110 | 0.0121 |
| Note. Models estimated using sample size of N=110 | | |

|  | | | | |
| --- | --- | --- | --- | --- |
| **Predictor** | **Estimate** | **SE** | **t** | **p** |
| Interceptᵃ | -0.0955 | 0.7590 | -0.126 | 0.900 |
| FEAR OF JUDGMENT FROM OTHERS | 0.0453 | 0.0894 | 0.507 | 0.613 |
| JUDGMENT PLEASANTNESS INTERACTION | 0.0798 | 0.1398 | 0.570 | 0.570 |
| OTHER PARTICIPANTS' ACQUAINTANCE |  |  |  |  |
| 1 – 0 | 0.2553 | 0.3472 | 0.735 | 0.464 |
| 2 – 0 | 0.2136 | 0.3454 | 0.619 | 0.538 |
| ᵃ Reference level | | | | |
