# Supplementary material for: Thinking together: How group argumentation boosts fake news recognition
Source: PLoS One. 2026 May 27;21(5):e0348391. doi: 10.1371/journal.pone.0348391 (PMC13215538; doi:10.1371/journal.pone.0348391)
Supplement: S1 Fig — (DOCX) [file pone.0348391.s005.docx]

**S1 Figure**

*Supplementary Figure s1. Distribution of accuracy scores for fake news by argumentation type and phase.*
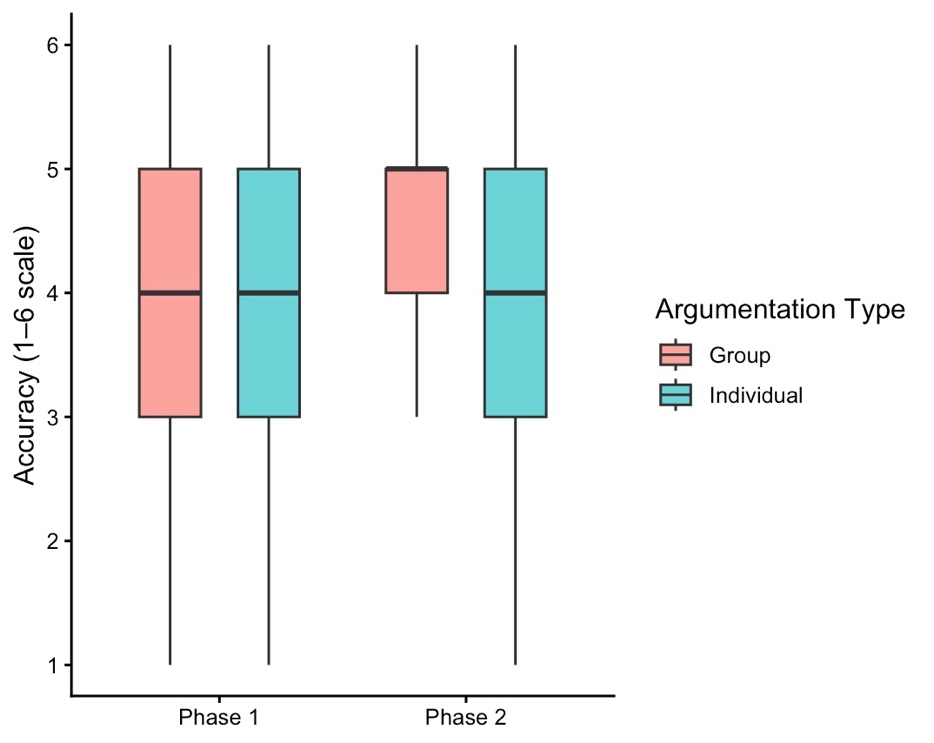


Boxplots showing the distribution of accuracy scores (1–6 scale) across phases (Phase 1 vs. Phase 2) and argumentation type (Individual vs. Group). Boxes represent the interquartile range, the horizontal line indicates the median, and whiskers extend to 1.5 × the interquartile range.
