# Supplementary figures and images for: Thinking together: How group argumentation boosts fake news recognition
Source: PLoS One. 2026 May 27;21(5):e0348391. doi: 10.1371/journal.pone.0348391 (PMC13215538; doi:10.1371/journal.pone.0348391)

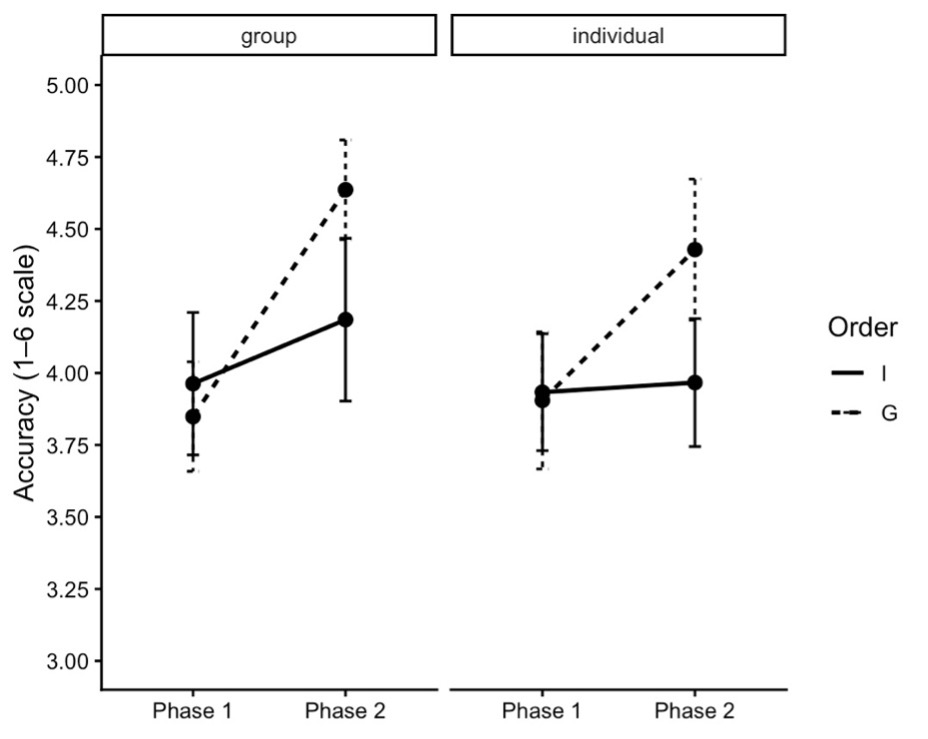

Supplement: S2 Fig — (JPG) [file pone.0348391.s006.jpg]

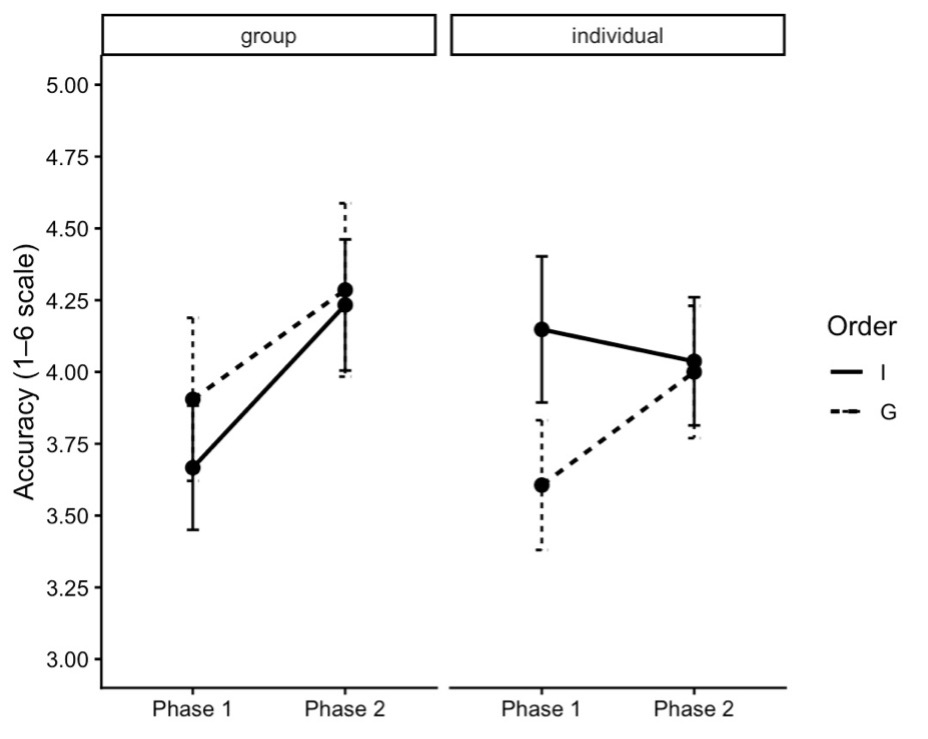

Supplement: S3 Fig — (JPG) [file pone.0348391.s007.jpg]
