# Supplementary material for: Thinking together: How group argumentation boosts fake news recognition
Source: PLoS One. 2026 May 27;21(5):e0348391. doi: 10.1371/journal.pone.0348391 (PMC13215538; doi:10.1371/journal.pone.0348391)
Supplement: S5 Fig — (DOCX) [file pone.0348391.s009.docx]

**S5 Figure**

Supplementary Figure s5. *Distribution of accuracy scores for thyroid by argumentation type, phase and order.*


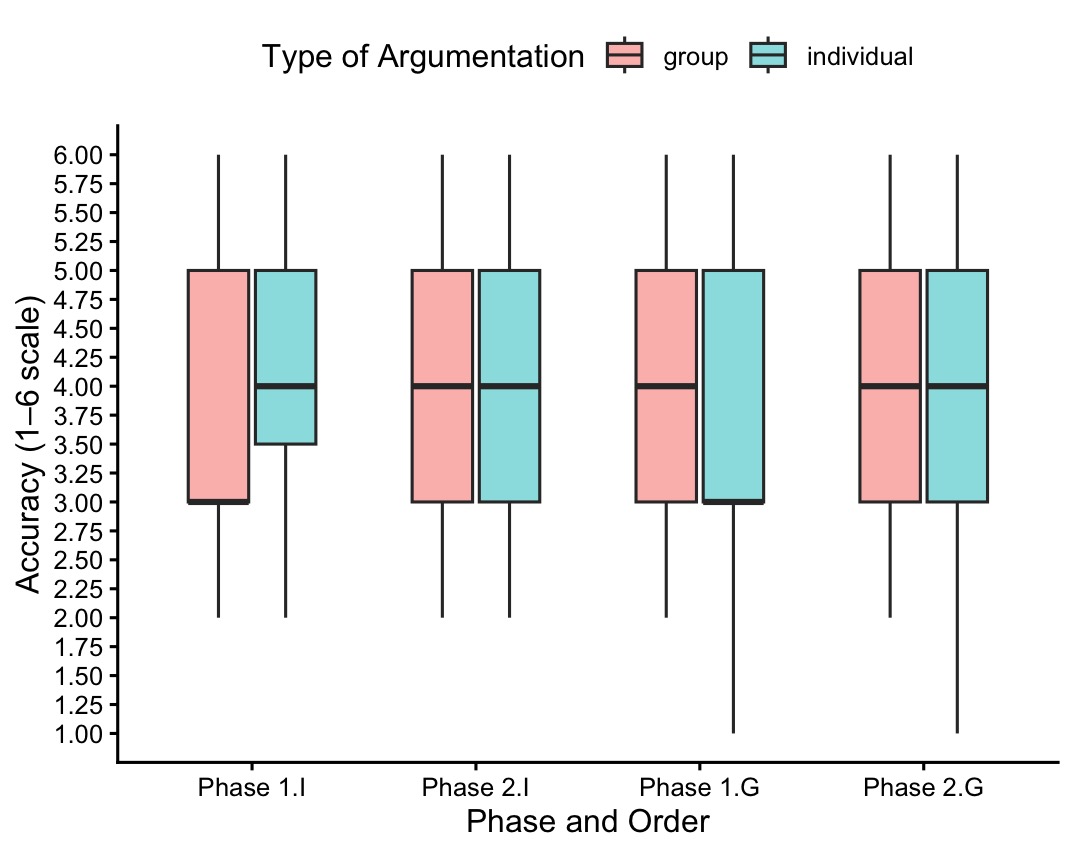


Boxplots show the distribution of accuracy scores for thyroid, separately for individually argued and group-discussed items across Phase 1 order I and G and Phase 2 order I and G. The boxplot indicates the median and interquartile range.
