# Supplementary material for: Thinking together: How group argumentation boosts fake news recognition
Source: PLoS One. 2026 May 27;21(5):e0348391. doi: 10.1371/journal.pone.0348391 (PMC13215538; doi:10.1371/journal.pone.0348391)
Supplement: Attachment — Submitted filename: Response to Reviewers.docx [file pone.0348391.s010.docx]

*EDITOR*

*Haixia Wang, Ph.D.*

*Academic Editor*

*PLOS ONE*

*Thank you for submitting your manuscript to PLOS ONE. After careful consideration, we feel that it has merit but does not fully meet PLOS ONE’s publication criteria as it currently stands. Therefore, we invite you to submit a revised version of the manuscript that addresses the points raised during the review process.*

Dear Editor, we thank you and the reviewers for the time and effort devoted to the evaluation of our manuscript entitled “Thinking Together: How Group Argumentation Boosts Fake News Recognition” (Manuscript ID: PONE-D-25-43739). We are pleased to submit a revised version of the manuscript, in which we have carefully addressed all the comments and suggestions provided during the review process.

As required, this response letter details how each comment has been addressed and how the manuscript has been revised accordingly. Our responses are provided in blue font below each comment.

***Additional Editor Comments:*** *This study has certain theoretical and practical implications, and there are still some methodological details that require modification:*

1. *The definition of fake news*
2. *The specific procedures of the experiment*
3. *Other issues mentioned by the two reviewers*

We thank the Editor for highlighting the need for greater conceptual clarity and methodological detail. In response, we have revised the manuscript to clarify the definition and use of the terms fake news and misinformation, and to ensure terminological consistency throughout the paper. In addition, we have expanded the description of the experimental procedures and addressed the methodological and interpretative issues raised by the reviewers. All changes are described in detail below and highlighted in the revised manuscript.

***Reviewer 1***

*The paper appears to adopt a novel approach; however, the author struggles to clearly define the concept of fake news. A precise definition has not yet been provided. Additionally, the author uses the term misinformation inconsistently, without establishing its contextual meaning, and shifts abruptly to disinformation, which carries a distinct implication. Therefore, it is suggested that the author refer to substantial prior works on the subject to develop clear conceptual distinctions among fake news, misinformation, and disinformation, and accordingly revisit and refine the literature review and data analysis, if necessary. They may refer/revisit works of
1. Wardle, C. Fake News (2017) It’s Complicated, First Draft News, https://firstdraftnews.com/fake-news-complicated/
2. European Commission Contribution to the European Council (2018) Action Plan Against
Disinformation. Brussels, 5 December, JOIN (2018) 36 final, https://ec.europa.eu/commission/
sites/beta-political/files/eu-communication-disinformation-euco-05122018_en.pdf/
3. UK, House of Commons, Digital, Culture, Media, and Sport Committee (2019) Disinformation
‘Fake News’: Final Report. https://publications.parliament.uk/pa/cm201719/cmselect/cmcumeds/1791/1791.pdf/
4. Commisso, C. (2017) ‘The post-truth archive: considerations for archiving context in fake news
repositories’, Preservation, Digital Technology and Culture, Vol. 46, No. 3, pp.99–102,
doi: https://doi.org/10.1515/pdtc-2017-0010.*

We thank the reviewer for highlighting the need to provide a clear definition of fake news and for emphasizing the importance of using related terms consistently throughout the manuscript. In response, we have added a dedicated paragraph in the Introduction clarifying how these terms are defined and used in the manuscript. We explicitly draw on prior influential work in the field (European Commission, 2018; UK & House of Commons Digital, Culture, Media, and Sport Committee, 2018; Wardle, 2017) as well as a recent systematic review providing a unified taxonomical framework (Kapantai et al., 2021). In particular, we now specify that we use the term fake news as a generic descriptor for news-like content containing false information, without reference to the intentionality of its source. Misinformation is used to refer in general to false or inaccurate information shared without deliberate intention to deceive. Terminology has been revised consistently throughout the manuscript, including the keywords and headings. References to disinformation have been removed, as the study does not address intentionality and the term was therefore not appropriate in this context.

Importantly, because the study does not assess or manipulate the intentionality behind the false information used as stimuli, these conceptual distinctions—while theoretically relevant—do not directly bear on the operationalization of the stimuli or on the analytical strategy adopted. We therefore believe that the main analyses and conclusions remain robust with respect to these distinctions. These revisions can be found in the Introduction (p. 3) and throughout the manuscript where terminology has been harmonized.

***Reviewer 2***

*This manuscript reports a within-subjects experiment (N = 111) examining whether group discussion enhances the ability to recognize fake news compared to individual written reflection. Participants judged the accuracy of two real and two fake news items in Phase 1, then re-evaluated the same items in Phase 2 after either a group discussion or an individual argumentation exercise. The key finding is that accuracy improved only for fake news discussed in groups, not for items argued individually. The authors also report a transfer effect: discussing one fake news item in a group increased accuracy for subsequently judged (but individually evaluated) fake news items.
The study is well motivated, conceptually interesting, and contributes to the literature on misinformation and the social dimension of reasoning. It addresses a theoretically meaningful question: Do group discussions actually make people better at detecting misinformation? The general pattern of results is clear and has implications for both theory and practice.
However, several issues limit the manuscript’s clarity, robustness, and generalizability. I outline these below.*

*We thank the reviewer for the careful and thorough evaluation of our manuscript. We greatly appreciate the detailed feedback, which has been extremely helpful in improving the clarity, rigor, and overall quality of the paper.*

*We have carefully considered each comment and observation and have made corresponding changes in the revised version of the manuscript. In the sections below, we provide a detailed, point-by-point explanation of how each observation has been addressed.*

***Major Comments***
*1. Limited number of stimuli
The study uses only four news items (two real, two fake). This severely restricts claim generalizability and inflates the influence of item idiosyncrasies (e.g., the “Nutella” real-news anomaly). Although the authors acknowledge this limitation, the problem is more serious than stated.
Recommendations:
• Treat results as preliminary; tone down conclusions accordingly.
• Include item-level analyses (e.g., mixed-effects models with random intercepts for items) if possible, even with small item numbers, to demonstrate the robustness of effects.
• Consider adding these data to a future preregistered replication with a substantially larger item pool.*

We thank the reviewer for these important observations regarding the limited number of news items used in the study. We agree that the small number of stimuli (two real and two fake news items) imposes constraints on the generalizability of the findings and may increase the influence of item-specific idiosyncrasies.

In response, we have taken several steps to address this concern:

1. Cautious framing of conclusions.
   We have revised the manuscript to adopt a more cautious tone. In particular, we have revised the Discussion by grounding our interpretations more explicitly in the relevant literature and by expanding the limitations section. We have also reviewed the wording of several statements throughout the manuscript. For example, the sentence in the Conclusions previously stating that the study “provides the first evidence in this direction” has been replaced with “provides preliminary evidence in this direction,” thereby more accurately reflecting the exploratory nature of the study.
2. Item-level analyses.
   To address the potential impact of item-related variability on our results, we removed the initial analysis that was intended to control for content effects (a 2 × 2 ANOVA by news content and phase, previously reported in the Supplementary Materials). We instead conducted linear mixed-effects models with phase as a fixed effect and news item as a random intercept to account for item-specific variability. No significant main effect of phase emerged, and item-level variance accounted for a notable proportion of the total variance (ICC = .125). When news type (fake vs. real) and its interaction with phase were included as fixed effects, the phase × news type interaction was significant, and item-level variance was substantially reduced (ICC = .029), indicating that most between-item variability was associated with news type rather than idiosyncratic properties of individual items (p. 10).
3. Future preregistered replication.
   We have also added a note in the discussion acknowledging that the study was exploratory and did not include preregistration. We suggest that future studies could replicate these findings with a larger pool of news items in a preregistered design to enhance robustness.

We believe these revisions address the reviewer’s concerns, clarify the exploratory scope of the study, and demonstrate that the observed effects are robust with respect to the idiosyncratic properties of individual items.

*2. Interpretation of “analytical thinking” is not directly supported
The manuscript repeatedly claims that group argumentation “stimulates analytical thinking” and that improvements occur because reasoning processes are activated. However, no measures of analytical thinking (CRT, need for cognition, verbal reasoning markers, etc.) are included.
Thus, this explanation is speculative.
Recommendations:
• Reframe claims to avoid strong causal statements about analytical thinking.
• Alternatively, incorporate linguistic analysis of written arguments (e.g., argument quality, evidence use) to support the interpretation.
• At minimum, clearly distinguish results from interpretation: the data show group-improved accuracy; the theoretical lens is Argumentative Theory*

We thank the reviewer for this important comment. By using the term analytical thinking, we intended to refer only to the idea that group discussion may foster a more critical evaluation of news content. However, we agree that this use of the term could generate ambiguity. Therefore, we have revised the manuscript to remove explicit references to analytical thinking throughout the text, including the Abstract and headings. The interpretation of the findings has been reframed within the framework of Argumentative Theory, which is used as a theoretical perspective to interpret the results rather than as a claim about specific underlying cognitive processes.

The revised manuscript more clearly distinguishes between the empirical findings—showing improved accuracy following group discussion—and their theoretical interpretation, thereby better aligning the scope of the conclusions with the available data.

*3. Methodological clarity in the group-discussion procedure
The instructions suggest that:
• Participants presented arguments one at a time, in experimenter-determined order,
• Followed by free discussion.
But several details are unclear:
• Did all groups actually use the full 6 minutes?
• Were experimenters present and visible?
• Were discussions recorded and coded? If not, why not?
• How were interruptions, dominance, or social loafing controlled for?
Because group dynamics are central to the claim, more detail is needed.
Recommendations:
• Provide more complete procedural information.
• Consider reporting descriptive features of groups (average talking time, whether all participants contributed, etc.), even qualitatively.*

We thank the reviewer for highlighting the need for more detailed information regarding the group-discussion procedure. In response, we have revised the Methods section to provide a clearer and more complete description of the procedure.

Specifically, we now clarify that:

- The group discussion began with a structured turn-taking phase, in which participants presented their arguments one after the other in the experimenter-determined order. This ensured that every participant had the opportunity to express their view, reducing potential effects of dominance, interruptions, or social loafing.
- The experimenter was visible only during the initial instruction phase, kept the camera off during the discussion, and intervened only when strictly necessary (e.g., in the event of prolonged silence).
- All members contributed at least once during the initial turn-taking phase.
- Almost all groups used the full 6 minutes allotted (range: 5 minutes 7 seconds to 6 minutes), with only one exception (4 minutes 27 seconds in a single discussion).
- All discussions were audio-recorded and subsequently transcribed in anonymized form. Recordings were deleted immediately after transcription.

These revisions provide more complete procedural transparency and address the reviewer’s concerns regarding the handling of group dynamics and the quality of participation.

4. *Statistical approach could be improved
   a. ANOVA may be suboptimal
   Given the within-subjects structure and non-independent items, a mixed-effects model (participants × items) would be more appropriate.
   b. Accuracy scores are ordinal
   A 1–6 scale is treated as continuous. This is common but should be acknowledged as a limitation.
   c. Multiple comparisons
   Several post-hoc tests are conducted. Corrections (Tukey is used in places, but inconsistently reported) should be applied consistently and transparently.*

We thank the reviewer for these thoughtful methodological comments, which helped us improve the clarity and robustness of the statistical analyses. We address each point in turn.

(a) Use of mixed-effects models.
We agree that mixed-effects models are appropriate given the within-subjects design and the non-independence introduced by group discussion. We addressed this concern in two complementary ways.

First, as reported in the Results section (p. 10), we conducted preliminary linear mixed-effects models including news item as a random intercept to assess the extent to which item-specific idiosyncrasies influenced the observed effects. These analyses showed that although item-level variance was present in a baseline model, it was largely explained by the distinction between fake and real news once news type and its interaction with phase were included. This supported our decision to aggregate accuracy scores by news type in the main analyses, while explicitly acknowledging the limited number of stimuli as a limitation in the Discussion.

Second, we conducted an additional robustness analysis for the effect of the type of argumentation using a linear mixed-effects model with random intercepts for group, reported in the Supplementary Materials (S2 File). Group was specified as a random effect to explicitly control for the possibility that the effect of argumentation type depended on systematic differences between groups (e.g., some groups being generally more accurate than others). By modeling group-level variance, we assessed whether belonging to a particular discussion group accounted for a substantial portion of the variance beyond the fixed effect of argumentation type. Although the intraclass correlation coefficient indicated non-negligible between-group variability, the phase × argumentation type interaction remained statistically significant, suggesting that the improvement observed for fake news discussed in groups cannot be attributed solely to group-specific characteristics.

This mixed-effects analysis was restricted to the first fake news item presented, in line with the study’s experimental aims. Because the study explicitly tested whether group discussion could influence subsequent individual evaluations of other fake news items, responses to later fake news items could plausibly reflect carry-over effects from earlier group discussion. Restricting the analysis to the first fake news item therefore allowed a more conservative test of the immediate effect of argumentation type, prior to any possible cross-item influence. Within this restricted dataset, each participant contributed to only one fake news observation; consequently, item could not be meaningfully specified as a random effect in this model. Concerns regarding item idiosyncrasies were addressed through the separate item-level mixed-effects analyses reported in response to Comment 1.

(b) Treatment of accuracy scores as continuous.
We acknowledge that accuracy was measured using a 1–6 Likert scale and treated as continuous in the analyses. While this practice is common and widely accepted in the literature, we now explicitly acknowledge it as a limitation in the Discussion and cite methodological work supporting and qualifying this approach.

(c) Multiple comparisons.
All post-hoc comparisons were conducted using Tukey HSD corrections. In the revised manuscript, we now report these comparisons consistently and transparently, explicitly stating that Tukey-adjusted post hoc tests were used and providing mean differences, standard errors, test statistics, and adjusted p values throughout.

*5. Order effects deserve deeper exploration
The “transfer effect” is one of the most interesting findings, but the interpretation is limited.
Questions needing clarification:
• Did the transfer effect occur for both fake items or only one?
• Could this be an artifact of stimulus difficulty order rather than group discussion?
• Did participants in the group-first condition also show more engagement or longer response times, suggesting increased task motivation rather than reasoning transfer?
Suggestions:
• Provide item-specific plots for each order condition.
• Analyze whether Phase 2 response times differ by order and argumentation type.
• Discuss alternative explanations more fully.*

We thank the reviewer for these thoughtful suggestions regarding the interpretation of the transfer effect and potential order-related confounds. We addressed these concerns in several ways.

First, to examine whether the transfer effect was driven by a specific fake news item, we now provide item-specific descriptive plots in the Supplementary Material. Separate graphs and boxplots are reported for the Formaldehyde and Thyroid fake news items across order conditions (S2–S5 Figures). Inspection of these plots reveals some item-specific differences. For example, accuracy judgments for the Thyroid fake news item in Phase 1 appear to be more sensitive to its position in the experimental sequence, with a tendency toward higher perceived veracity when the item appears later. Importantly, however, despite these item-specific patterns, the order-related improvement associated with the group-first condition is observable for both fake news items, indicating that the transfer effect does not depend on the idiosyncratic behavior of a single stimulus.

Second, regarding the possibility that the transfer effect reflects stimulus difficulty or presentation order rather than group discussion, we clarified in the Results section that the fake news items were presented using a partially counterbalanced design. Each fake news item was discussed in a group by half of the participants and argued individually by the other half. This design feature rules out the possibility that the observed transfer effect is driven by the intrinsic content or difficulty of a specific fake news item.

The reviewer also suggested examining Phase 2 response times as a potential indicator of increased engagement or task motivation. Response times were not recorded in this study because in tasks requiring the production of open-ended argumentative texts, response times are strongly influenced by individual and situational factors (e.g., writing speed, planning strategies, self-monitoring) and are therefore often only weakly informative about task difficulty or depth of cognitive processing.

However, we considered the helpful suggestion and assessed whether the transfer effect could be explained by increased motivation or engagement rather than by reasoning-related mechanisms. To this end, we conducted additional analyses using the number of words written in individual arguments as a proxy for engagement, a measure commonly used as an index of cognitive or motivational investment in prior research (as in Kuhn et al., 2024; Kuhn & Modrek, 2021). A factorial nonparametric analysis (ART) revealed a significant Order × News Type interaction. Follow-up analyses showed that, in the group-first order, the increase in argument length was statistically significant for real news—where no accuracy transfer effect was observed—but not for fake news, where the transfer effect was present. This dissociation suggests that increased engagement, operationalized as argument length, is unlikely to fully account for the observed improvement in fake news accuracy (pp. 13-14).

Finally, we expanded the Discussion to more explicitly consider alternative explanations for the transfer effect and to clarify why a purely motivational account does not sufficiently explain the observed pattern of results.

*6. Potential confounds in Phase 2
Participants saw the same news items again. This raises at least two concerns:
a. Memory effects
Accuracy improvements could reflect remembering earlier judgments.
b. Familiarity effects
Prior exposure may reduce belief in real news (a known finding) and can increase belief in fake news (illusory truth effect). The study acknowledges neither.
While the authors argue that individual reflection does not cause improvement, the design still conflates repeated exposure with deliberation.
Recommendation:
• Include a discussion of familiarity and exposure effects as alternative mechanisms*

We thank the reviewer for raising this important concern regarding potential memory and familiarity confounds associated with repeated exposure to the same news items in Phase 2.

In response, we have added a dedicated paragraph to the Discussion explicitly addressing memory effects, familiarity effects, and the illusory truth effect as alternative explanatory mechanisms. We now acknowledge that repeated exposure could, in principle, influence accuracy by facilitating memory for earlier judgments or by increasing familiarity with the headlines.

However, we also clarify why these mechanisms are unlikely to account for the observed pattern of results. Prior research shows that repeated exposure often increases perceived accuracy of fake news through the illusory truth effect (e.g., Fazio et al., 2022; Pennycook et al., 2018; Pennycook & Rand, 2021). In contrast, in the present study repeated exposure was associated with a reduction in belief in fake news, and this improvement occurred selectively following group discussion, rather than uniformly across phases or conditions. If familiarity or exposure per se were driving the effect, one would typically expect either no change or an increase in perceived accuracy of fake news across conditions.

We further note that familiarity effects are attenuated when individuals actively evaluate the accuracy of information at initial exposure (Calvillo & Smelter, 2020), and that repetition does not reliably produce familiarity-driven backfire effects when evaluative or corrective processes are engaged (Ecker et al., 2023). These considerations suggest that the improvement observed in Phase 2 cannot be straightforwardly attributed to memory or familiarity effects.

Finally, while we acknowledge that the design necessarily involves repeated exposure and deliberation, the condition-specific nature of the accuracy gains—emerging only after group discussion and not following individual reflection—indicates that repeated exposure alone is insufficient to explain the results. We have revised the Discussion to more explicitly consider these alternative mechanisms and to clarify why a purely exposure-based account is unlikely.

*7. Social factors may be under-examined
The authors report that social variables (fear of judgment, familiarity with group members, enjoyment of discussion) did not predict gains. But:
• The items used to measure these constructs are single-item measures with unknown reliability.
• The study does not examine whether certain group compositions (e.g., mixed-accuracy triads) produce more benefit.
Given the strong role of group quality in predicting improvement, more attention to social processes seems warranted.*

We thank the reviewer for these important comments regarding the social-emotional variables (fear of judgment, familiarity with group members, enjoyment of the interaction). We acknowledge that these constructs were assessed using single-item measures, which limits reliability. For this reason, we do not interpret these measures as definitive indicators of social processes, but rather as exploratory controls aimed at assessing whether simple social-interaction factors could account for the observed accuracy gains. We have clarified this point in the Discussion and adopted more cautious language when interpreting these null effects. Moreover, we have clarified in both the Methods and Discussion that the experimental procedure was designed to minimize other social confounds. The structured turn-taking phase ensured that all participants contributed at least once, reducing the impact of dominance, interruptions, or social loafing, and the experimenter did not actively participate in the discussion.

Importantly, in response to the reviewer’s suggestion to further examine group composition, we conducted an additional analysis focusing on pre-discussion dissent within groups, reported in the Results section (p. 17). Groups were categorized as heterogeneous, F-homogeneous (all members initially judged the fake news as false), or T-homogeneous (all members initially judged the fake news as true). This analysis revealed no significant interaction between group type and accuracy improvement across phases. Notably, from a descriptive perspective, even in T-homogeneous groups—where all members initially endorsed the incorrect interpretation—nearly half of participants shifted toward the correct answer following group discussion, rather than exhibiting polarization toward the incorrect response. This pattern suggests that the benefits of group discussion are not limited to specific group compositions and are not driven solely by initial majority accuracy.

We conclude that our results do not rule out the importance of social processes per se, but rather emphasize the role of the social dimension of reasoning in providing a better understanding of the observed effects.

***Minor Comments***

*1. Pre-registration
There is no mention of preregistration. Because the analytic choices (e.g., averaging across items) are somewhat flexible, preregistration would strengthen the contribution.*

We thank the reviewer for raising this point. The study was not preregistered, as it was designed as an exploratory investigation aimed at identifying potentially relevant patterns rather than testing a fully specified set of a priori hypotheses. As the reviewer correctly notes, some analytic decisions (e.g., the aggregation of items) involved a degree of flexibility. We have now explicitly acknowledged the absence of preregistration as a limitation of the study and noted that future confirmatory research would benefit from preregistered analytic plans to further strengthen the robustness of the findings.

*2. Figures could be clearer
o CI bars overlap in many places, making visual interpretation difficult.
o Consider adding raw distribution plots or violin/boxplots.*

We thank the reviewer for this helpful suggestion. To improve visual clarity and address the issue of overlapping confidence intervals, we added boxplots to the Supplementary Material (Figures S1, S4, S5), which provide a clearer visual summary of accuracy scores across conditions.

*3. Terminology:
The term “phase” is accurate but could be more explicitly defined earlier in the manuscript.*

We thank the reviewer and, in line with their suggestion, we have revised the initial description of the procedure to explicitly define the two experimental phases at their first introduction. In the revised version, we now clearly state that the within-subjects design included two main phases (Phase 1 and Phase 2), improving terminological clarity.

*4. Stimuli selection:
The rationale for choosing specific real/fake items could be more thoroughly justified beyond simple pretesting.*

We thank the reviewer for this helpful comment. We have expanded the Stimuli section to clarify the rationale underlying the selection of the real and fake news items beyond pretesting. In the revised manuscript, we now specify that stimuli were selected to ensure diversity in topic, temporal relevance, and salience, and that all items were required to contain debatable content. News items merely reporting isolated factual events were excluded, as they offer limited opportunity for argumentation or discussion. This clarification highlights how stimuli selection was guided by the study’s theoretical focus on deliberation and group discussion.

*5. Generalizability:
The sample (mostly young Italian students, 79% female) limits applicability to other populations; this should be highlighted.*

We thank the reviewer for this important observation. We have now expanded the Discussion to more explicitly acknowledge the limited generalizability of the findings. In particular, we note that the sample consisted of university students, a population likely familiar with collaborative group work, that it may not include individuals most susceptible to misinformation, and that the sample was predominantly female (79%). We also emphasize that future research should examine whether the observed effects extend to other populations and settings.

***Overall Evaluation*** *This is an interesting paper with a clear result: group argumentation improves fake news detection more than individual reflection does. The study aligns with findings from Argumentative Theory and meaningfully extends the literature by applying group-reasoning paradigms to misinformation.
However, the manuscript requires:
• clearer methodological detail,
• more cautious interpretation,
• a more thorough exploration of alternative explanations,
• improved statistical framing, and
• stronger discussion of limitations.
With revisions, the manuscript has potential to contribute meaningfully to the field.*

We thank the reviewer for this overall evaluation of the manuscript. We carefully considered all the points raised and revised the paper accordingly, with the aim of improving methodological clarity, interpretative caution, and the discussion of alternative explanations and limitations. We are sincerely grateful for the reviewer’s thoughtful and detailed comments, which contributed substantially to improving the overall quality and clarity of the manuscript.

We hope that these revisions adequately address the reviewers’ and editor’s concerns, and we thank them again for their constructive and insightful feedback.

**References**

Calvillo, D. P., & Smelter, T. J. (2020). An initial accuracy focus reduces the effect of prior exposure on perceived accuracy of news headlines. *Cognitive Research: Principles and Implications*, *5*(1), 55. https://doi.org/10.1186/s41235-020-00257-y

Ecker, U. K. H., Sharkey, C. X. M., & Swire-Thompson, B. (2023). Correcting vaccine misinformation: A failure to replicate familiarity or fear-driven backfire effects. *PLOS ONE*, *18*(4), e0281140. https://doi.org/10.1371/journal.pone.0281140

European Commission (Ed.). (2018). *A multi-dimensional approach to disinformation: Report of the independent High level Group on fake news and online disinformation*. Publications Office. https://doi.org/10.2759/0156

Fazio, L. K., Pillai, R. M., & Patel, D. (2022). The effects of repetition on belief in naturalistic settings. *Journal of Experimental Psychology: General*, *151*(10), 2604–2613. https://doi.org/10.1037/xge0001211

Kapantai, E., Christopoulou, A., Berberidis, C., & Peristeras, V. (2021). A systematic literature review on disinformation: Toward a unified taxonomical framework. *New Media & Society*, *23*(5), 1301–1326. https://doi.org/10.1177/1461444820959296

Kuhn, D., Bruun, S., & Geithner, C. (2024). Enriching Thinking Through Discourse. *Cognitive Science*, *48*(3), e13420. https://doi.org/10.1111/cogs.13420

Kuhn, D., & Modrek, A. (2021). Mere exposure to dialogic framing enriches argumentive thinking. *Applied Cognitive Psychology*, *35*(5), 1349–1355. https://doi.org/10.1002/acp.3862

Pennycook, G., Cannon, T. D., & Rand, D. G. (2018). Prior exposure increases perceived accuracy of fake news. *Journal of Experimental Psychology: General*, *147*(12), 1865–1880. https://doi.org/10.1037/xge0000465

Pennycook, G., & Rand, D. G. (2021). The Psychology of Fake News. *Trends in Cognitive Sciences*, *25*(5), 388–402. https://doi.org/10.1016/j.tics.2021.02.007

UK, & House of Commons Digital, Culture, Media, and Sport Committee. (2018). *Disinformation and ‘fake news’: Final Report*. https://publications.parliament.uk/pa/cm201719/cmselect/cmcumeds/1791/1791.pdf

Wardle, C. (2017, February 16). *Fake news. It’s complicated.* First Draft News. https://firstdraftnews.org/articles/fake-news-complicated/
