# Supplementary material for: Thinking together: How group argumentation boosts fake news recognition
Source: PLoS One. 2026 May 27;21(5):e0348391. doi: 10.1371/journal.pone.0348391 (PMC13215538; doi:10.1371/journal.pone.0348391)
Supplement: Attachment — Submitted filename: Response_to_Reviewers_auresp_2.docx [file pone.0348391.s011.docx]

*EDITOR*

*Haixia Wang, Ph.D.*

*Academic Editor*

*PLOS ONE*

Dear Editor,

We would like to thank you and the reviewers for the time and effort devoted to the evaluation of our manuscript entitled “T*hinking Together: How Group Argumentation Boosts Fake News Recognition*” (Manuscript ID: PONE-D-25-43739R1). We are pleased to submit a revised version of the manuscript, in which we have addressed the additional comments and suggestions provided during the latest round of review.

As requested, this response letter details how each comment has been addressed and how the manuscript has been revised accordingly. Our responses are provided in blue font below each comment, and all modifications in the manuscript are highlighted using track changes.

***Editor Comments***

*The paper closely engages with the theoretically and practically significant question of whether group argumentation aids in the identification of fake news, addressing a gap in current misinformation research concerning the social dimension of cognition. However, the following issues still require consideration:*

*(1) Sample representativeness remains limited.
The sample consists primarily of Italian university students, with a disproportionately high percentage of female participants (79%), which may affect the generalizability of the findings. It is recommended that this limitation be more explicitly acknowledged in the discussion, and that future research validate the findings across more diverse populations.*

We appreciate the editor’s comment regarding sample representativeness. The limitations section now explicitly acknowledges the limited representativeness of the sample. We clarified that the sample consisted of university students and that the majority of participants were female (79%), which may limit the representativeness and generalizability of the findings. We also noted that future research should examine whether the results extend to more diverse populations and settings.

*(2) The number of stimulus items is still limited (four news items).
Although the authors used mixed-effects models to control for content heterogeneity, it remains difficult to fully rule out the influence of item-specific characteristics. It is recommended that the conclusions continue to emphasize the preliminary nature of the findings, and that future studies expand the stimulus set.*

We agree with the editor that the limited number of stimuli should be explicitly acknowledged. The limitations section now notes that the study relied on a small number of stimuli (four news items). We also clarified that future studies should expand the stimulus set in order to further examine the robustness and generalizability of the findings. In addition, the concluding paragraph emphasizes the preliminary nature of the results within a tightly controlled experimental setting, albeit with a limited set of stimuli.

*(3) The mechanism underlying the "transfer effect" requires further investigation.*

*The study found that individuals became more accurate in judging another fake news item after engaging in group discussion—a highly interesting transfer effect. However, its underlying mechanism remains unclear (e.g., transfer of cognitive strategies vs. increased motivation). It is recommended that this effect be interpreted more cautiously in the discussion, and that future research incorporate mediating variables (such as argument quality or changes in cognitive strategies) to further examine the mechanisms at play.*

We thank the editor for this comment. In the discussion section we now explicitly acknowledge that the mechanism underlying the transfer effect remains unclear and that further research is needed to examine it. We have therefore added a sentence suggesting that future research should incorporate mediating variables (e.g., argument quality or changes in cognitive strategies) to further investigate the mechanisms underlying this effect.

***Reviewer 1***

*Following the discussion section, a conclusion may be included highlighting the key contributions and major findings of the study, along with suggestions and directions for future research.*

We appreciate the reviewer’s suggestion. The manuscript now includes a concluding paragraph summarizing the main findings and highlighting the implications of structured group discussion for improving misinformation detection. We also outline directions for future research, including the need to examine whether the results generalize to more diverse populations and contexts, to further investigate the pattern observed in the present study that is consistent with a transfer effect, and to explore potential mediating variables (such as argument quality) as well as possible social influences (for example, by comparing exposure to written arguments with active participation in group discussions).

***Reviewer 2***

*The authors have responded thoroughly and in good faith to the previous round of major revisions. The manuscript is substantially improved, and the additional analyses, procedural clarifications, and expanded discussion largely address my earlier concerns. I thank the authors for the care and seriousness with which they engaged with the review.*

*Before final acceptance, I recommend a small number of minor, editorial revisions, focused on interpretive clarity rather than additional analyses:*

We are very pleased with the reviewer’s positive assessment and would like to thank them once again for their thoughtful comments, which have helped us improve the manuscript substantially. We respond to the additional comments below.

1. *Generalisability and robustness.
   Although the manuscript now appropriately acknowledges the limited number of stimuli and adopts a more cautious tone overall, there are still a few places where the language implies a level of generality or robustness that exceeds what can reasonably be inferred from a four-item design. I recommend ensuring that claims, particularly in the Abstract, Conclusions, and discussion of mixed-effects results, are consistently framed as preliminary and specific to the present paradigm.*
2. *Framing of the transfer effect.
   The additional analyses and item-level plots strengthen the case that the observed order-related improvement is not driven by a single stimulus. However, the transfer effect remains interpretive rather than definitive. I suggest maintaining consistent caution in how this effect is described (e.g., “consistent with” or “suggestive of” transfer) across the Results and Discussion.*
3. *Scope clarification.
   A brief, explicit sentence clarifying the scope of the findings, for example, that they demonstrate the potential benefits of structured group discussion within a tightly controlled, small-stimulus experimental context, would further help align conclusions with the evidential base.*

We thank the reviewer for these helpful suggestions. Following the reviewer’s recommendation, we revised the wording throughout the manuscript to ensure that the findings are consistently framed in a cautious manner and interpreted as preliminary within the context of a small-stimulus experimental design. In particular, we softened several formulations in the Abstract, Results, and Discussion (e.g., replacing “evidence that group discussion improves” with “preliminary evidence that group discussion may improve”, and describing the observed pattern as “consistent with a transfer effect” rather than implying a definitive transfer mechanism). We also revised the concluding paragraph to clarify the scope of the findings and to emphasize that they derive from a tightly controlled experimental setting albeit with a limited set of stimuli.

We hope that the revisions adequately address the concerns raised during the review process, and we would like to thank the editor and the reviewers again for their constructive feedback.
